# Global fjords as transitory reservoirs of labile organic carbon modulated by organo-mineral interactions

**DOI:** 10.1126/sciadv.add0610

**Published:** 2022-11-18

**Authors:** Xingqian Cui, Alfonso Mucci, Thomas S. Bianchi, Ding He, Derrick Vaughn, Elizabeth K. Williams, Chuning Wang, Craig Smeaton, Katarzyna Koziorowska-Makuch, Johan C. Faust, Alain F. Plante, Brad E. Rosenheim

**Affiliations:** ^1^ School of Oceanography, Shanghai Jiao Tong University, Shanghai, China.; ^2^ GEOTOP and Earth and Planetary Sciences, McGill University, Montreal, Quebec, Canada.; ^3^ Department of Geological Sciences, University of Florida, Gainesville, FL, USA.; ^4^ Department of Ocean Science and Hong Kong Branch of the Southern Marine Science and Engineering Guangdong Laboratory (Guangzhou), The Hong Kong University of Science and Technology, Hong Kong, China.; ^5^ Department of Earth and Environmental Science, University of Pennsylvania, Philadelphia, PA, USA.; ^6^ School of Geography and Sustainable Development, University of St Andrews, St Andrews, UK.; ^7^ Institute of Oceanology, Polish Academy of Sciences, ul. Powstańców Warszawy 55, Sopot, Poland.; ^8^ MARUM-Center for Marine Environmental Sciences, University of Bremen, Leobener Strasse 8, 28359 Bremen, Germany.; ^9^ College of Marine Science, University of South Florida, St. Petersburg, FL, USA.

## Abstract

The global carbon cycle is strongly modulated by organic carbon (OC) sequestration and decomposition. Whereas OC sequestration is relatively well constrained, there are few quantitative estimates of its susceptibility to decomposition. Fjords are hot spots of sedimentation and OC sequestration in marine sediments. Here, we adopt fjords as model systems to investigate the reactivity of sedimentary OC by assessing the distribution of the activation energy required to break OC bonds. Our results reveal that OC in fjord sediments is more thermally labile than that in global sediments, which is governed by its unique provenance and organo-mineral interactions. We estimate that 61 ± 16% of the sedimentary OC in fjords is degradable. Once this OC is remobilized and remineralized during glacial maxima, the resulting metabolic CO_2_ could counterbalance up to 50 ppm of the atmospheric CO_2_ decrease during glacial times, making fjords critical actors in dampening glacial-interglacial climate fluctuations through negative carbon cycling loops.

## Teaser

The labile nature of sedimentary organic carbon makes fjords critical actors in the global carbon cycle.

## INTRODUCTION

The evolution of the global climate is strongly governed by the cycling of carbon (*1*, *2*). At the Earth’s surface (the exogenic cycle), CO_2_ is released from anthropogenic activities (e.g., fossil fuel burning and cement production), volcanic eruptions, metamorphism, and the oxidative weathering of rock-derived organic carbon (OC_petro_) and carbonates, when the latter is fueled by the oxidation of sulfide minerals (*3*–*5*). This is counterbalanced by CO_2_ drawdown through silicate weathering and burial of biospheric OC (OC_bio_) (*3*, *6*, *7*). Because oxidative weathering of sedimentary rock and silicate weathering are closely coupled to long-term climate change (~10^6^ to 10^7^ years) and mountain building (*1*, *2*), OC biosynthesis and its ultimate burial in sedimentary basins serve as efficient processes to balance the exogenic carbon budget over glacial-interglacial time scales (i.e., 10^4^ to 10^5^ years) (*3*, *7*). Therefore, a thorough understanding of the carbon-climate feedback requires quantitative estimates of OC fluxes between various carbon pools (*3*, *6*).

The balance between OC sequestration and decomposition is critical in modulating the carbon-climate feedback (*3*, *4*, *6*–*9*). As the largest active carbon pool on Earth, the global ocean is estimated to bury 160 Tg of OC year^−1^ in marine sediments (*10*, *11*), with small changes responsible for regulating the global atmospheric composition and climate regime. However, OC burial rates vary spatially across marine depositional systems, with areas adjacent to marginal seas generally being carbon burial hot spots (places where disproportionally high amounts of OC are buried per unit area) (*9*, *12*). With 90% of sedimentary OC burial occurring in the ocean, marginal seas play a disproportionate role in OC sequestration and climate regulation (*10*, *13*). In comparison, OC decomposition, as the primary determinant of carbon burial efficiency, is initiated shortly after biosynthesis and continues through low- and high-temperature diagenesis, metamorphism, and respiration upon exhumation (*2*–*4*, *14*). Over geological time scales, hot spots of OC burial can equally be hot spots of carbon decomposition (*4*, *13*) and thus serve as ideal systems to examine the balance between OC burial and decomposition. However, external forces (e.g., glaciers) may additionally disturb the OC burial-decomposition balance.

Numerous studies have reinforced the notion that fjords are hot spots for carbon burial (*12*, *15*–*17*), but little is known about the susceptibility of this OC pool to degradation (*18*, *19*). Fjords are often valley-type inlets of the sea carved by advancing and retreating glaciers at glacial periods, whereas others may arise from tectonic activity (*20*). Following postglacial sea level rise, fjords often become semiconnected to the open ocean and serve as depocenters for terrigenous and marine-derived OC (*21*). With an estimated OC burial rate of 18 Tg year^−1^, fjords store 11 to 12% of the total OC buried in the global ocean over 10^4^-year time scales despite covering only ~0.1% of the ocean surface area (*12*, *17*). During glacial periods, interglacial sediment deposits in fjords can be remobilized and redeposited as morainal sills near their outer reaches or on the adjacent shelves by advancing glaciers (*22*). Accordingly, OC buried below the sediment oxygen penetration depth in fjords during interglacial periods can potentially be decomposed after being reexposed to O_2_ during remobilization (*12*, *21*). Thus, a thorough understanding of OC reactivity and potential mechanisms affecting OC decomposition is a prerequisite for a quantitative assessment of the decomposable OC in fjords and to understand the role of fjords in glacial-interglacial carbon-climate feedbacks.

In this study, we examined the reactivity of sedimentary OC from 25 fjords to elucidate the potential vulnerability of this large OC pool to decomposition in the coastal ocean. By comparing the reactivity of sedimentary OC sampled at various locations worldwide, along with data gathered from the literature, we attempt to assess the spatial variability of sedimentary OC reactivity in fjords. By exploring mechanisms that control sedimentary OC reactivity in fjords, we further estimate the fraction and total quantity of OC that can be readily remineralized in fjord sediments. Given the unique glacially carved nature of many fjords and their glacial-interglacial geomorphological and carbon cycling features, relative to other coastal ocean systems (*21*), we show evidence that fjords, by hosting a transitory pool of thermally labile OC, may serve as critical thermostats of climate fluctuations.

### Characterizing sedimentary OC reactivity

The reactivity of sedimentary OC is generally defined as its susceptibility of being decomposed, biotically or abiotically (*23*–*26*). Sedimentary OC is either physiochemically bound to mineral surfaces, trapped within particle mesopores, or unprotected as particulate organic debris (*14*, *27*, *28*), contributing to the diverse range of OC reactivities. In general, “bulk” geochemical analyses characterize the average composition of all OC contained within a sample but offer limited information on the reactivity of the complex sedimentary mixture (*8*, *9*). Conversely, biomarkers, such as amino acids and lignin, target less than 1% of the total OC pool (*29*). Hence, whereas biomarkers may provide clues on the reactivity of the OC, the interpretations can be heavily biased because bulk OC consists of a complex mixture of compounds (*13*, *24*).

Thermal decomposition of organic matter (OM) bridges the gaps in the information gained from bulk OC and biomarker analyses (Materials and Methods). Ramped combustion–evolved CO_2_ gas analysis (RC-EGA) and ramped pyrolysis oxidation–^14^C (RPO-^14^C) analysis techniques can assess the susceptibility of OC to decomposition upon exposure to continuously ramped-up temperatures (*23*–*25*, *30*, *31*). Once carbon bonds are broken, volatile OC fragments are oxidized to CO_2_ downstream (*24*, *30*). The strength of the OC bonds, either intramolecular or inter–organo-mineral, reflects the resistance of OM to thermal decomposition and, presumably, its reactivity, which, in turn, serves as a proxy of OC lability to biogeochemical decomposition (*26*). Hence, the distribution of OC bond strengths, as characterized by their activation energy (*E*) [termed p(*E*)] (table S1) (*24*), reflects the diversity of OC lability within a complex mixture. As quantitative measures, μ*_E_* is defined as the mean, whereas σ*_E_* is defined the SD, of the p(*E*) (*23*, *24*).

RPO-^14^C analyses were also carried out (Materials and Methods), whereby evolved CO_2_ is collected as four fractions spanning different temperature ranges. Fraction-specific stable carbon (δ^13^C) and radiocarbon [^14^C activity, reported as fraction modern (Fm)] isotopic measurements performed on CO_2_ fractions offer insights on carbon lability controlled by carbon sources and transit times (*23*, *30*, *32*). Note that thermal *E*, as used here, represents the modeled energy required to break OC bonds via thermochemical decomposition under the parameters (e.g., Arrhenius pre-exponential factor) imposed to the inversion model. Therefore, the value of thermal *E* depends on the model parameters and is not equivalent to the activation energy of any natural enzymatic respiration reaction (*23*, *26*). Thus, our interpretation of OC reactivity relies solely on relative *E* values and previously reported thresholds, not on absolute values of specific samples.

## RESULTS AND DISCUSSION

### Spatial heterogeneity of OC reactivity in fjords

**Fig. 1.**
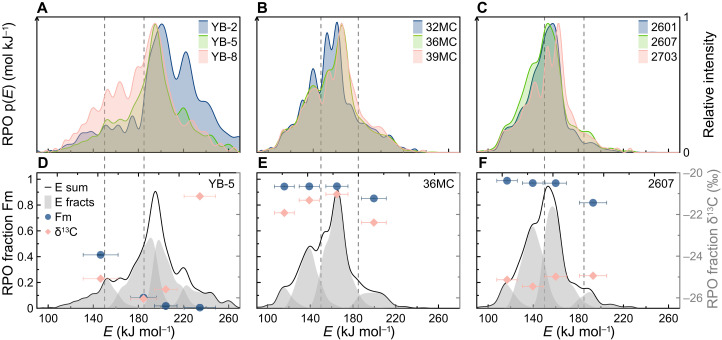
The distribution of activation energy [p(*E*)] along selected fjord proximal-to-distal transects and the corresponding fraction split–based activation energy distributions and isotopic compositions from RPO-^14^C analysis. (**A**) The p(*E*) value of fjord proximal (YB-2), middle reach (YB-5), and distal (YB-8) stations in Yakutat Bay, Southeast Alaska. (**B**) The p(*E*) value of fjord proximal (32MC), middle reach (36MC), and distal (39MC) stations in Sitka Sound, Southeast Alaska. (**C**) The p(*E*) value of fjord proximal (2601), middle reach (2607), and distal (2703) stations in Dusky Sound. The fraction-specific p(*E*) distributions (four gray-shaded areas), δ^13^C values, and Fm values of three samples from the middle reaches of (**D**) Yakutat Bay (YB-5), (**E**) Sitka Sound (36MC), and (**F**) Dusky Sound (2607). Note that each sample is split into four fractions, as annotated with four gray-shaded areas (E fracts), dark blue dots (Fm), and pink diamonds (δ^13^C) in (D) to (F). The isotopic signatures (δ^13^C and Fm) of fraction splits were corrected for the activation energy distribution. Horizontal error bars represent the SD values of *E* for split fractions. The dashed vertical lines represent low-*E*, mid-*E*, and high-*E* boundary splits, whereas the black curves in (D) to (F) represent the p(*E*) (E sum) of the bulk sample.

To evaluate the influence of local spatial variability on OC reactivity, we first examined the thermal decomposition characteristics of sedimentary OC recovered along three fjord proximal-to-distal transects (fig. S1A), where the primary sources of OC are contrastingly different. Yakutat Bay (YB-2, YB-5, and YB-8; fig. S1B), Sitka Sound (32MC, 36MC, and 39MC; fig. S1C), and Dusky Sound (2601, 2607, and 2703; fig. S1D) were selected as representative fjords dominated, respectively, by petrogenic OC (OC_petro_), marine OC (OC_mari_), and terrestrial OC (OC_terr_) inputs. In practice, the relative proportions of these three types of OC differ along fjord transects and between geographic regions (*18*, *33*, *34*). On the fjord-specific level, OC properties/reactivities shift toward lower *E* and *T*_max_ (temperature of the maximum CO_2_ evolution) values along the proximal-to-distal transect in the OC_petro_-dominated fjord (Yakutat Bay; Fig. 1 and fig. S2), which is likely attributed to inputs of labile OC_mari_ in the distal reaches (*16*, *34*). In contrast, reversed proximal-to-distal *E* and thermogram distribution patterns are observed in fjords dominated by OC_bio_ (i.e., OC_mari_ and OC_terr_) (Fig. 1 and fig. S2), owing mainly to the degradation and enhanced mineral protection of sedimentary OC during along-axial transport (*35*, *36*). Overall, our *E* distributions corroborate previous reports of proximal-to-distal patterns of OC content, isotopic signatures, and biomarkers in fjords (*15*, *16*, *21*, *34*, *37*). Nevertheless, despite the spatial variability of OC sources and their degradation states, the thermal reactivity of samples from the middle reaches is constrained well within the range of fjord distal and proximal sediments and is most representative of the mean activation energy (μ*_E_*) of sedimentary OC in each fjord (Fig. 1).

**Fig. 2.**
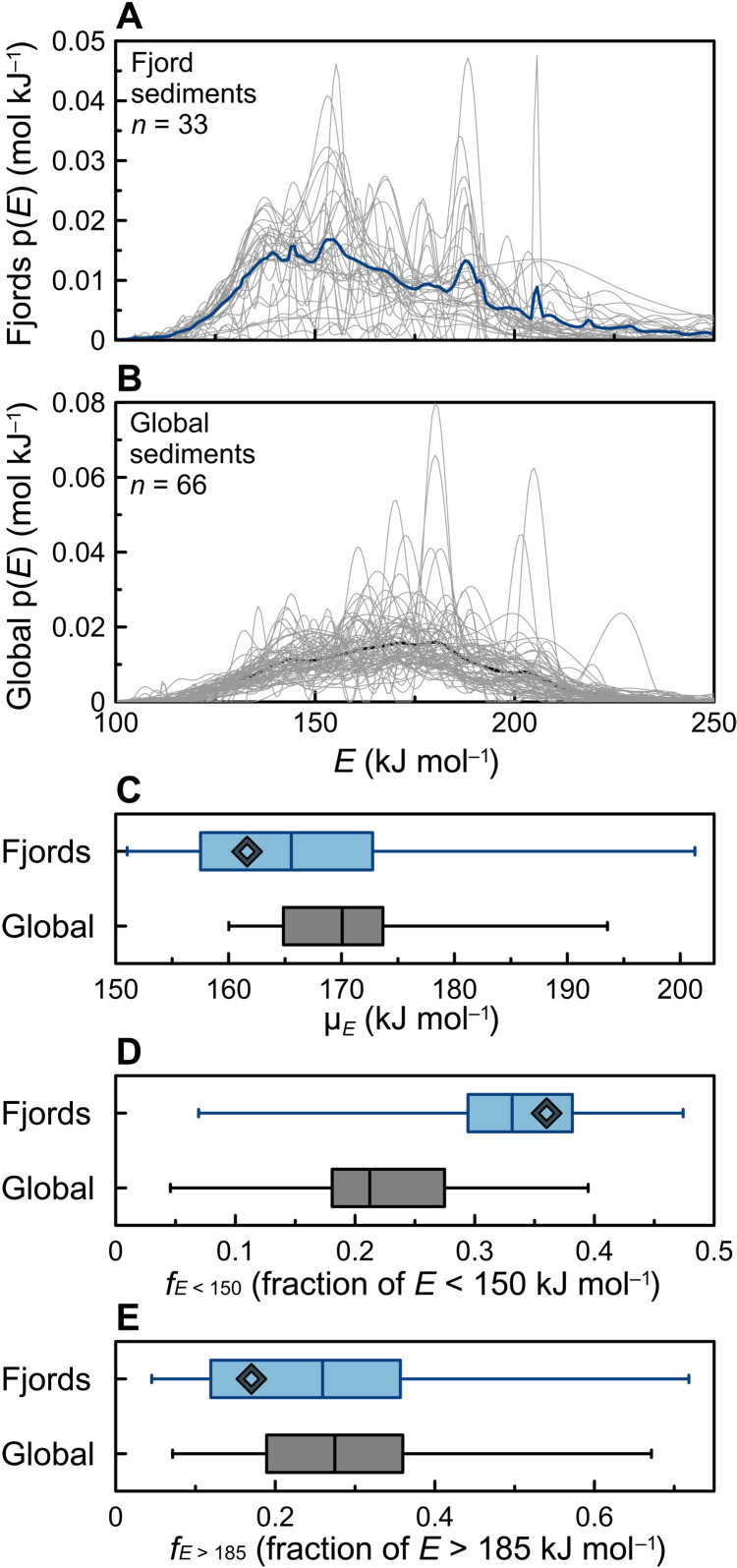
A comparison of activation energy distribution [p(*E*)] and *E*-based fractions between fjord sediments and the global sediment compilation. (**A**) The p(*E*) value of fjord sediments from thermogravimetric infrared analysis in this study. Each gray curve represents 1 sample, whereas the dark blue curve is the average p(*E*) value of all 33 fjord samples. (**B**) The updated p(*E*) value of the global sediment compilation based on the dataset in (*23*) (Supplementary Materials). Note that gray and black curves represent individual samples and sample averages, respectively. (**C**) Box-whisker plot of mean *E* values (μ*_E_*) for fjord sediments and the global sediment compilation. Comparisons of OC fractions with (**D**) *E* < 150 kJ mol^−1^ and (**E**) *E* > 185 kJ mol^−1^ between fjord sediments and the compiled global sediments, presented as box-whisker plots. The black diamonds in (C) to (E) represent fjord average values. In (C) to (E), the boxes represent quartiles, whereas whiskers indicate the minimum/maximum values.

Given that the middle reach best represents the mean features of OC activation energy in each fjord, we extended our investigation to sediments from the middle reaches of an additional 22 large fjords from around the world (fig. S1 and table S2). Overall, the activation energy of fjord OC spans large ranges with diverse p(*E*) variabilities between samples (Fig. 2A). The μ*_E_* value of sedimentary OC in fjords, derived from our sample set, ranges between 151.0 and 201.3 kJ mol^−1^(Fig. 2C and table S3), which is slightly more variable than the range of global sedimentary OC (160.0 to 193.6 kJ mol^−1^) (*23*). Other than the wider ranges, fjord OC p(*E*) peaks cluster at *E* ≍ 155 and 188 kJ mol^−1^ (Fig. 2A), of which the bimodal p(*E*) feature is distinct from the global sediment single peak at *E* ≍ 170 to 182 kJ mol^−1^ (Fig. 2B) (*23*).

### OC reactivity modulated by OC provenance and OC-mineral interactions

We suggest that the bimodal distribution of p(*E*) (Fig. 2A) is likely driven by the intrinsic properties of various OC sources, revealed by notable differences in major p(*E*) windows and fraction-specific isotopic signatures in fjords with dissimilar OC inputs. For example, the majority of OC in the OC_petro_-dominated fjord (i.e., Yakutat Bay) yields an *E* value between 168 and 240 kJ mol^−1^ [p(*E*) > 10^−2^ mol kJ^−1^] or thermally decomposes between 400° and 700°C (Fig. 1 and fig. S2). This contrasts with the range of values {*E* = 110 to 174 kJ mol^−1^ [p(*E*) > 10^−2^ mol kJ^−1^] or 300° to 450°C} observed in fjords dominated by OC_mari_ (i.e., Sitka Sound) and OC_terr_ (i.e., Dusky Sound), which are indistinguishable from each other based on their *E* distributions and thermograms. Furthermore, the first fraction collected following thermal decomposition at lower temperatures (fractional μ*_E_* = 147 kJ mol^−1^) of the OC_petro_-dominated fjord sediments contains relatively greater Fm and δ^13^C values than the higher-temperature fractions with fractional μ*_E_* ≥ 185 kJ mol^−1^ (table S4), indicative of a young, labile OC_mari_ contribution in the lower *E* spectrum (Fig. 1) (*38*, *39*). In contrast, the highest-temperature fractions, in fjords dominated by OC_mari_ (fractional μ*_E_* = 200 kJ mol^−1^) and OC_terr_ (fractional μ*_E_* = 193 kJ mol^−1^), yield significantly lower Fm values than the other fractions with fractional μ*_E_* ≤ 165 kJ mol^−1^ (*P* < 0.01) (Fig. 1 and table S4), a feature that we tentatively attribute to the dominance of OC_petro_ or inputs of preaged, refractory OC_terr_ (*32*). Thus, the bimodal peaks of p(*E*) at ≍155 and 188 kJ mol^−1^ likely correspond to the primary decomposition windows of young, thermally labile OC_bio_ and OC_petro_, respectively, highlighting the bipolar provenance of OC in global fjords.

**Fig. 3.**
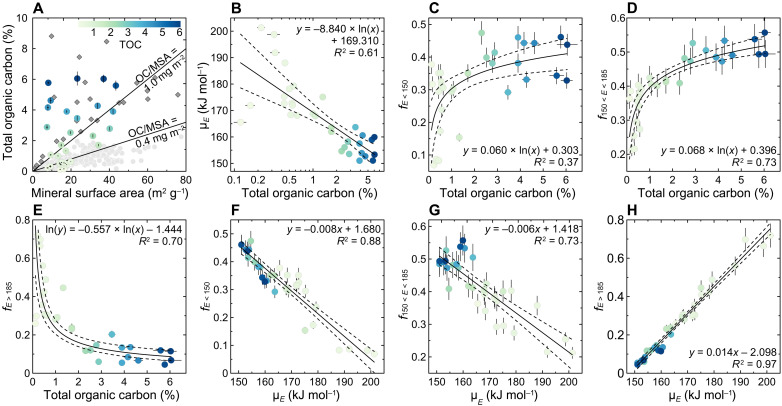
Correlations of total OC content, OC loading (OC/MSA), mean activation energy (μ*_E_*), fractions of OC with *E* < 150 kJ mol^−1^ (*f*_*E* < 150_), 150 < *E* < 185 kJ mol^−1^ (*f*_150 < *E* < 185_), and *E* > 185 kJ mol^−1^ (*f*_*E* > 185_). (**A**) The correlation of total OC content and MSA of fjord sediments (colored dots), Clayoquot Sound, Vancouver Island, British Columbia (gray diamonds) (*17*), and sediments from typical passive margins (light gray dots) (*13*, *43*). Two solid lines represent the OC/MSA thresholds of 0.4 and 1.0 mg of OC m^−2^. (**B** to **E**) Correlations of total OC content versus μ*_E_*, *f*_*E* < 150_, *f*_150 < *E* < 185_, and *f*_*E* > 185_. (**F** to **H**) Correlations of μ*_E_* versus *f*_*E* < 150_, *f*_150 < *E* < 185_, and *f*_*E* > 185_. Solid and dashed lines in (B) to (H) are regression curves and 95% CI, respectively. All correlations are statistically significant (*P* < 0.01). Note that the *x* axis in (B) is presented on a log scale. The colored bar and dots represent the range of total OC contents.

**Fig. 4.**
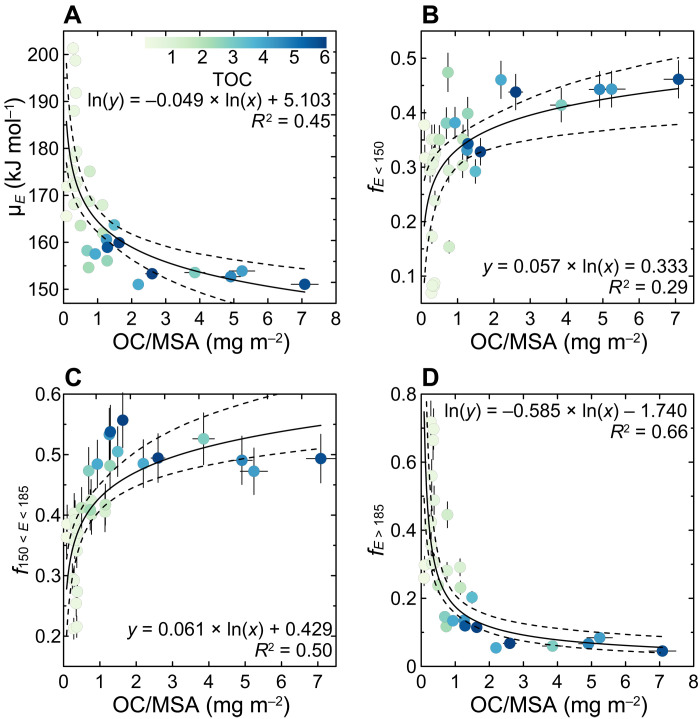
Correlations of OC loading (OC/MSA) with mean activation energy (μ*_E_*) and fractions of OC with *E* < 150 kJ mol^−1^ (*f*_*E* < 150_), 150 < *E* < 185 kJ mol^−1^ (*f*_150 < *E* < 185_), and *E* > 185 kJ mol^−1^ (*f*_*E* > 185_). Cross-plots of OC/MSA with (**A**) μ_E_, (**B**) *f*_E < 150_, (**C**) *f*_150 < E < 185_, and (**D**) *f*_E > 185_. The colored bar and dots show the range of total organic carbon (TOC) values, as illustrated in Fig. 3. Solid and dashed lines are the best fit curves and 95% CI, respectively.

In comparison, the observed μ*_E_* variability and variable p(*E*) distributions are attributed to the mineral protection of OC. Despite reasonably accounting for the bimodal p(*E*) feature, bipolar OC input functions, based on their distinctive p(*E*) peak windows, do not fully explain the wide range of μ*_E_* or justify the p(*E*) distributions in the spatially diverse fjords examined in this study. Secondary p(*E*) peaks, observed between 155 and 180 kJ mol^−1^ (Fig. 2A), are inconsistent with typical thermal features of either OC_petro_ or young, labile OC_bio_. Although each type of OC may yield specific *E* peaks, they typically integrate a broad range of *E* value as they interact with various mineral particles and form aggregates (*23*, *25*, *39*–*41*). Accordingly, we propose that the observed variability of μ*_E_* and the diverse p(*E*) distributions reflect variable levels of mineral protection, largely modulated by the abundance of OC relative to the availability of mineral surfaces [or mineral surface area (MSA)] and their chemical interactions (*27*, *42*). Along ocean margin shelves and deltas, sedimentary OC content (%OC) is typically strongly correlated to MSA, with relatively constant OC/MSA (OC loading) values (*13*, *42*–*46*). In comparison, %OC is unrelated to MSA in our dataset (Fig. 3A), suggesting that MSA does not control %OC in fjords. Previous studies found that the %OC in fjords is instead controlled by mineral dilution (*15*, *20*). As expected, values of μ*_E_* correlate significantly with %OC [coefficient of determination (*R*^2^) = 0.61; *P* < 0.01; Fig. 3B] and OC/MSA (*R*^2^ = 0.45; *P* < 0.01; Fig. 4A) of fjord sediments, underscoring the dependency of carbon reactivity on the “richness” of OC relative to minerals in fjord sediments. Overall, it suggests that mineral dilution determines the OC content and OC/MSA, which further controls p(*E*) distributions and μ*_E_* values in global fjord sediments.

The isotopic composition of various *E* intervals provides additional constraints to distinguish the pools of OC and thus their interactions with minerals. Fraction-specific μ*_E_* and carbon isotopic signatures (δ^13^C and Fm) reflect the substantial inputs of young OC_bio_ in *E* ≤ 147 (YB-5-1; or fraction 1 of YB-5; same as below), *E* ≤ 160 (2607-3), and *E* ≤ 165 kJ mol^−1^ (36MC-3), whereas OC_petro_ and refractory, preaged OC predominates when *E* ≥ 185 (YB-5-2), *E* ≥ 193 (2607-4), and *E* ≥ 200 kJ mol^−1^ (36MC-4; Fig. 1, D to F, and table S4). Thus, values of *E* = 150 and 185 kJ mol^−1^ appear to be valid thresholds to distinguish between relatively young and thermally labile, intermediate, and highly refractory OC in sediments, corroborating previous assertions (*32*, *38*). Sensitivity tests further show that our findings are not sensitive to the selected thresholds, as the correlation coefficients between fractions of OC and other parameters (e.g., OC/MSA and %OC) were examined by shifting thresholds by ±10 kJ mol^−1^ (i.e., 140 to 160 and 175 to 195 kJ mol^−1^; fig. S3). Accordingly, bulk OC was separated into three subcomponent fractions based on low *E* values of <150 kJ mol^−1^ (*f*_*E* < 150_), mid *E* values between 150 and 185 kJ mol^−1^ (*f*_150 < *E* < 185_), and high *E* values of >185 kJ mol^−1^ (*f*_*E* > 185_) (table S1).

Our results reveal that organic debris (aggregates or large pieces of OM derived from the vegetation or soil) dominates the more reactive fractions. The proportion of OC with low *E* (*f*_*E* < 150_; Materials and Methods; Eq. 1) correlates positively with %OC (*R*^2^ = 0.37; *P* < 0.01; Fig. 3C) and OC/MSA (*R*^2^ = 0.29; *P* < 0.01; Fig. 4B) in fjord sediments. The curved shifts of *f*_*E* < 150_ at higher %OC and OC/MSA values suggest that OC-rich samples consist primarily of reactive OC with lower activation energies, likely composed of organic debris free of mineral interactions (*18*, *23*, *32*, *39*). For example, OC-rich sediments (%OC > 1%) typically display OC/MSA values exceeding the threshold of 1 mg m^−2^ of the “patchy” OC coating model (Fig. 3A) (*8*, *42*, *45*). Accordingly, much of the OC would be unbound to mineral surfaces, a common feature in highly productive regions where the total sedimentary OC content exceeds the maximum mineral protection capacity (*8*, *13*, *43*). Furthermore, *f*_*E* < 150_ and σ*_E_* values fluctuate within narrow ranges at higher %OC values and OC/MSA (Figs. 3 and 4 and fig. S4), implying that the overall reactivity of the sedimentary OC becomes nearly invariant as it is progressively dominated by free (little or no mineral surface binding) organic debris (*23*, *47*).

Conversely, the high-*E* fraction of OC (*f*_*E* > 185_) is negatively correlated with %OC (*R*^2^ = 0.70; *P* < 0.01; Fig. 3E) and OC/MSA (*R*^2^ = 0.66; *P* < 0.01; Fig. 4D) and exceeds 0.5 in samples with low %OC (<0.5%) and OC/MSA (<0.4 mg m^−2^) values (Figs. 3 and 4). Thus, when MSA is abundant relative to the OC content, as indicated by the low OC/MSA ratios, OC can interact more effectively with mineral surfaces through absorption or mesopore inclusion (*27*, *43*). Furthermore, given that high-*E* fractions are composed of a mixture of OC_petro_ and partially preaged OC_terr_ (Fig. 1, D to F), much greater activation energies possibly arise from the fact that the OC_petro_ and a fraction of preaged OC_terr_ are also trapped within the mineral interlamellar space (*14*), making them even more recalcitrant to decomposition. This hypothesis is supported by the results of studies that show the OC content of Cretaceous black shales and modern sediments to be closely related to the mineral interlayer surface area and smectite content (*14*, *48*).

The mid-*E* (150 < *E* < 185 kJ mol^−1^) fraction that represents a smaller fraction of the OC in fjords than in global sediments exhibits strong positive correlations with %OC and OC/MSA (Figs. 3 and 4), alluding to a similar provenance as the OC characterized by low-E fractions. The highest *f*_150 < *E* < 185_ values are observed in samples with intermediate OC/MSA values and are primarily found in the fjords of New Zealand, southern Southeast Alaska, and British Columbia (Fig. 4, fig. S1, and table S3), where mid-*E* fractions display relatively high Fm values (Fig. 1). Therefore, the predominance of mid-*E* fractions originating primarily from OC aging can be ruled out (*39*). As samples with OC dominated by *f*_150 < *E* < 185_ also have larger σ*_E_* values (fig. S4), we attribute the mid-*E* predominance to variable levels of organo-mineral interactions (*23*, *27*). Thus, the mid-*E* fraction most likely consists of refractory macro-organic debris, OC bound to mineral surfaces through various mechanisms (e.g., ligand exchange, ion exchange, and van der Waals forces), isolated in mesopores, and trapped in mineral interlayer spaces (*25*, *27*, *28*, *47*, *49*). The mid-*E* fraction may additionally include substrates chemically altered by enzymatic digestion (*32*, *50*). The predominance of mid-*E* fractions is also observed in preaged soil OC, riverine particulate OC, and sedimentary OC from passive margin systems (*25*, *39*, *41*, *47*, *50*), of which the latter is primarily composed of preaged OC that interacts with minerals after thousands of years of winnowing (*8*, *39*, *40*, *43*).

In summary, young, labile OC_bio_, either as debris or loosely bound to minerals, makes up most of the low-*E* and mid-*E* fractions, whereas lithogenic, bedrock-derived refractory OC_petro_ predominates the high-*E* window. Furthermore, whereas μ*_E_* is strongly and negatively correlated to *f*_*E* < 150_ (*R*^2^ = 0.88; *P* < 0.01; Fig. 3F) and *f*_150 < *E* < 185_ (*R*^2^ = 0.73; *P* < 0.01; Fig. 3G), the correlation is positive with *f*_*E* > 185_ (*R*^2^ = 0.97; *P* < 0.01; Fig. 3H). This illustrates a strong dependence of μ*_E_* on the thermochemical diversity of OC in fjord sediments, driven by the overall strength of OC-mineral interactions. Thus, it seems reasonable to conclude that, in general, the level of organo-mineral interaction governs the mean reactivity of OC in contemporary fjord sediments.

### Quantitative estimates of OC reactivity

**Fig. 5.**
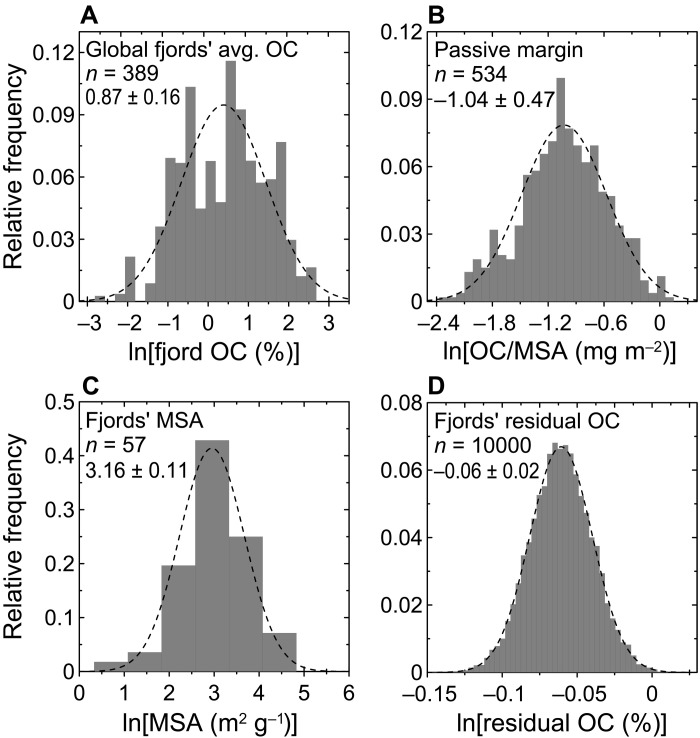
A visualization of compiled datasets and modeled ROC in fjord sediments, presented in natural log scales. (**A**) The OC content distribution of 389 worldwide fjord sediments from updated datasets in (*12*, *15*) (see the Supplementary Materials), whereas the mean and SD values of the ln(%OC) were calculated on the basis of the 1 × 10^4^ times of bootstrap resampling. (**B**) The compiled OC loading (OC/MSA) along passive margins from updated datasets in (*13*, *43*, *46*) (see the Supplementary Materials). (**C**) MSA data compiled from this and a previous study (*17*). (**D**) The estimated glacial OC content in fjord sediments after reaching a steady-state level (termed “residual OC”), based on 1 × 10^4^ times of bootstrap resampling. Note that (A) to (C) present compiled raw datasets, whereas (D) exhibits modeled results. Values in each subplot are given in natural log values. Dashed curves are the best fits to the data. Data are presented as relative frequencies.

Our results demonstrate that OC in fjord sediments is more reactive than that in global sediments. To quantitatively estimate the OC reactivity in fjords, we calculated the average μ*_E_* and proportions of *f*_*E* < 150_ and *f*_*E* > 185_ at the global scale. Unfortunately, these cannot be achieved by simply averaging values derived from this study, because the arithmetic mean derived from a limited dataset is heavily biased by sample distribution and representativeness. Therefore, we approached this problem by first adopting an updated %OC dataset of fjord sediments (*12*) that now comprises 389 measurements and applied a bootstrapping resampling (1 × 10^4^) on a per-fjord basis (Materials and Methods). The %OC data in fjords follow a logarithmic normal distribution with the mean and SD of ln(%OC) being 0.87 and 0.16, which correspond to %OC value of 2.42 ± 0.39% (Materials and Methods; Eqs. 2 to 4), and a 95% confidence interval (CI) of 1.74 to 3.28% (Eq. 5 and Fig. 5A). This result corresponds to an average OC μ*_E_* value of 161.6 ± 1.4 kJ mol^−1^ for global fjord sediments, as determined from the correlation between %OC and μ*_E_* (Fig. 3B). The average fjord OC μ*_E_* value is significantly lower (*P* < 0.001) than the global sediment average of 169.8 ± 7.2 kJ mol^−1^ (Fig. 2C) (*23*). Regressions between proxies allow further estimates of *f*_*E* < 150_ and *f*_*E* > 185_ values. The average value of *f*_*E* < 150_, based on its regression with %OC (Fig. 3C), is predicted to be 0.36, identical to the estimate of 0.36 projected from the average μ*_E_* value (Fig. 3F). Thus, the proportion (0.36) of low-*E* (*f*_*E* < 150_) sedimentary OC in fjords is significantly greater (*P* < 0.001) than in global sediments (~0.22 ± 0.07) (Fig. 2D), bolstering the assertion that sedimentary OC stored in fjords is relatively thermally labile.

The inferred reactivity and limited mineral association of OC in fjords are consistent with sediment dynamics in these depositional environments. Given the source attribution of the low-*E* OC component to unbound, labile OC debris, the presence of a large amount of unprotected organic debris is promoted by the steep topography of fjord valleys, frequent mass wasting events along fjord banks, rapid fluvial transport of organic debris, turbidity currents, and the persistence of steep salinity gradients and strongly stratified water columns (*12*, *17*, *18*, *34*, *36*). OC transit times in these systems rarely exceed 10^3^ years (fig. S5) (*18*, *34*), much shorter than the thousands of years along passive margin fluvial systems (*8*, *40*, *43*, *50*).

The global average *f*_*E* > 185_ value of sedimentary OC in fjords is estimated to be 0.19, based on its regression with μ*_E_* value (Fig. 3H), and 0.14 when plotted against %OC (Fig. 3E). Consistent with our finding that the sedimentary OC is highly labile in fjords, the average proportion of high-*E* OC (0.17 ± 0.03) in fjords, obtained by averaging values (0.14 and 0.19) generated from the above two methods, is slightly less but not statistically significantly different from that in global sediments (0.28 ± 0.14) (Fig. 2E). High-*E* fractions in typical marginal sea sediments are primarily composed of preaged OC_terr_ and OC_petro_ (*30*, *32*, *38*, *39*, *50*), because extensive reworking of OC_bio_ in these systems appears to provide sufficient time for sedimentary OC to bind tightly with minerals. Conversely, limited reworking of sedimentary OC and organo-mineral interactions in fjords facilitates the chemical decomposition of OC_bio_ in the low-*E* and mid-*E* windows, leaving the high-*E* fractions primarily composed of OC_petro_ (Fig. 1). Note that *f*_*E* > 185_ is not equivalent to the OC_petro_ fraction because OC_petro_ may also be liberated in the mid-*E* fraction once it is chemically altered (*32*) and that a small fraction of OC_bio_ is liberated within the high-*E* window. Hence, it is reasonable to surmise that fjords may host a considerable fraction of OC_petro_ (*18*, *33*, *34*) that may have originated from the erosion of carbonaceous bedrock in fjord catchment basins.

### A transitory pool of labile carbon storage in fjords

Fjords, formed during glacial periods and filled with thermochemically reactive OC during the interglacial stages, likely act as climate stabilizing capacitors. Sediment ages in contemporary fjords rarely exceed 2 × 10^4^ years (*20*, *22*), suggestive of efficient excavation of predeposited sediments in most fjords by advancing glaciers. Through the excavation process, a substantial fraction of the OC buried during interglacial times can be remobilized and exposed subaerially for thousands of years during glacial periods, making the most labile OC vulnerable to decomposition, mimicking processes occurring along extensive passive margin systems. Glacial dredge deposits have been observed worldwide near fjord mouths and adjacent shelves (*22*, *51*), and remobilized morainal sills are typically organic lean (<1%) compared with mid-fjord sediments (*17*, *52*, *53*), potential evidence of sedimentary OC decomposition during glacial remobilization.

Given the results of this study and the preceding discussion, we propose a framework to quantitatively estimate the amount of decomposable versus refractory OC in fjord sediments (Materials and Methods; Eq. 6). Whereas unbound organic debris is prone to aerobic decomposition, mineral-protected OC is assumed to be largely resistant to remineralization (*8*, *54*). Because organo-mineral interactions serve as a primary carbon protection mechanism in fjord sediments and MSA exerts a first-order control on sedimentary OC contents after extensive reworking, carbon loading (OC/MSA) may provide a tangible estimate of the postglacial remnant OC content in fjord sediments. Carbon loading has been similarly used to estimate OC mineralization and burial efficiency over multiple time scales in marginal sea systems (*8*, *10*, *43*, *44*, *55*).

In the case of fjords, there are two key factors to consider: the ultimate carbon loading after extensive aerobic degradation and the available MSA of fjord sediments. Carbon loading in fjord sediments after glacial reworking may be comparable to loading in systems where similarly prolonged OC degradation occurs. Passive margin systems may be suitable analogs because OC may experience thousands of years of winnowing and aerobic degradation before burial (*6*, *8*), ideal analogs to estimate postglacial carbon loading in fjords. Carbon loading data compiled for passive margin systems display a logarithmic normal distribution with an average value of 0.40 ± 0.20 mg m^−2^ (Fig. 5B and fig. S5). With 60% of the compiled data being lower than 0.4 mg m^−2^ (fig. S5), higher OC/MSA values are observed in the outer shelves, where local primary production may elevate sedimentary OC loading (*43*). This is consistent with the proposed threshold of 0.4 mg m^−2^ used to distinguish relatively stable organo-mineral association from the residual background state after prolonged aerobic decomposition (*8*, *43*, *44*). The projected OC loading in fjords may be an upper limit because many high-latitude, glaciated fjords exhibit OC/MSA < 0.4 mg m^−2^ (Figs. 3 and 4 and table S3). MSA data are scarce for fjord sediments. Nevertheless, on the basis of previously published data (Fig. 3A) (*17*) and data acquired in this study (table S3), the MSA of fjord sediments displays a logarithmic normal distribution and is estimated to be 23.8 ± 2.7 m^2^ g^−1^ (Fig. 5C).

Accordingly, the postdegradation mean OC content in fjord sediments is estimated at 0.94 ± 0.02%, with a 95% CI of 0.90 to 0.98% (Fig. 5D; Materials and Methods; Eqs. 7 and 8), which may be a conservative estimate when considering the potential lower OC loadings in some fjords. The estimated postglacial sedimentary OC contents in fjords are further corroborated by comparable %OC values reported for many riverine, deltaic, and continental margin systems (*13*, *43*, *44*), in accordance to the rationale of our model estimates. Our calculation suggests that ~61 ± 16% of the OC buried in fjord sediments is easily degradable (Materials and Methods; Eqs. 9 and 10), approximating the low-*E* and half of the mid-*E* fractions. Given constant OC accumulation rates and assuming a burial rate of 18 × 10^12^ g of OC year^−1^ for global fjords (*12*), a 10^4^-year sediment archive would preserve 11 × 10^16^ ± 3 × 10^16^ g of degradable OC in fjords. Assuming that this degradable OC in global fjords is remobilized and remineralized during glacial maxima and that this CO_2_ is added back to the atmosphere, it would be equivalent to an increase of 51 ± 14 parts per million by volume (ppm_v_) in atmospheric CO_2_. Nevertheless, further verification would likely decrease the estimated uncertainty of this amount.

These quantitative estimates provide a theoretical basis to illustrate a unique OC cycling pattern in fjords. In contrast to the common glacial/interglacial cyclic pattern of atmospheric *p*CO_2_ (*4*), our results suggest that fjords may potentially release a substantial amount of CO_2_ through OC remineralization during glacial maxima, corroborating largely variable radiocarbon ages (*56*) and lower δ^13^C values (*57*) of the atmospheric carbon reservoir during the last glacial period. If true, it may suggest that fjords were CO_2_ sources, counterbalancing approximately 50 ppm_v_ of the atmospheric CO_2_ drawdown, during glacial times. Consequently, by releasing CO_2_ during glacial times and absorbing CO_2_ during interglacial times, fjords may mitigate *p*CO_2_ fluctuations during glacial-interglacial cycles. Hence, the described OC burial-decomposition cycle determines the uniqueness of fjords as marine sedimentary OC reservoirs (*21*).

Note that the contribution of fjords to climatic regulation through OC cycling is not unique. Extensive marginal sea systems, once they approach a steady-state OC-mineral binding status after extensive predepositional degradation and reworking (*8*, *43*, *46*), have also been proposed as a consistent CO_2_ source during glacial periods, driven by pyrite oxidation (*4*). Likewise, given the considerable store of sedimentary carbonates in some fjords (*58*), the dissolution of carbonates during glacial maxima, when promoted by pyrite oxidation, may amplify the significance of fjords in climate regulation. CO_2_ release to the atmosphere through various processes during glacial times may be a common feature of glacial cycles, a component that has been mostly ignored in the past. The overall amount of CO_2_ released to the atmosphere during glacial times and its contribution to climatic regulation have yet to be determined, but our results suggest that many systems may interact with each other and cofunction to dampen glacial-interglacial climate swings through negative climate feedbacks (*59*).

## MATERIALS AND METHODS

### Samples

A total number of 33 surface sediment samples were collected from 25 fjords in Greenland (2), Svalbard (4), Northern Europe (3), Southeast Alaska (6), Eastern Canada (1), Western Canada (1), Patagonia (4), New Zealand (3), and Antarctica (1) (table S2). Twenty-five samples are from the middle reach of each fjord, whereas the remaining eight samples originate from three fjord proximal-middle-distal transects of the Yakutat Bay, Sitka Sound, and Dusky Sound. After freeze-drying, a fraction of each sample was saved for sediment MSA analysis, whereas the rest was homogenized for bulk measurements. An aliquot of each homogenized sample was further washed with hydrochloric acid to remove carbonates, freeze-dried, and homogenized for thermochemical analysis.

### Mineral surface area

Approximately 0.3 to 0.5 g of each freeze-dried sediment sample was placed in an oven and baked at 350°C for 24 hours to remove most of the OM (*43*). A higher combustion temperature was avoided, as it may alter mineral surface and porosity structures (*46*). Roughly 0.1 to 0.2 g of OM-free sediments were weighed in a sample port that was loaded into a Micromeritics TriStar II 3020 surface area analyzer. After being outgassed for 0.5 hours at 200°C to remove water (*46*), the specific MSA of the sediment was measured following the 10-point Brunauer-Emmett-Teller method (*43*). The average SD of the MSA, based on duplicate measurements, is estimated to be 1.7%.

### OC content and δ^13^C measurements

An aliquot of freeze-dried, homogenized sediment containing ~200 μg of OC was weighed and placed in a silver capsule. After adding a drop of ultrapure water and being fumigated in a desiccator with 12 molar HCl, samples were dried overnight in the oven at 45°C. Each silver capsule containing the decarbonated sediment samples was further wrapped in a tin capsule and loaded onto the autosampler of a Carlo Erba 2500 CN elemental analyzer coupled to a Thermo Fisher Scientific Electron DELTA V Advantage isotope ratio mass spectrometer (*15*, *34*). The OC contents and δ^13^C values were measured against U.S. Geological Survey 40 and 41 standards. The δ^13^C values were normalized to the Vienna Pee Dee belemnite scale. Precisions of the OC content and δ^13^C measurements, based on duplicate measurements, were <5% (relative) and 0.1 per mil (‰; absolute), respectively.

### RPO-^14^C analysis

The design and operation of the in-house assembled RPO-^14^C system were described in detail previously (*24*, *30*, *60*). Briefly, the RPO-^14^C system is sequentially made up of a carrier gas supply unit, programmable pyrolysis ovens, an infrared CO_2_ analyzer, switchable cryogenic traps, and a vacuum line. Throughout the analysis, a constant flow of helium (35 ml min^−1^) is maintained through the pyrolytic furnace, and a mixture of helium and oxygen (helium, 7 ml min^−1^; oxygen, 4 ml min^−1^) is supplied directly through the lower chamber. Ten decarbonated, freeze-dried sediment samples were selected for RPO analysis. Sediments containing approximately 1 mg of OC or <330 mg of mass, whichever was less, were loaded in a quartz reactor that was introduced into the pyrolytic chamber of the upper pyrolysis oven. Samples were subjected to pyrolysis from ambient temperature to 900° to 1000°C at a constant ramping rate of 5°C min^−1^. In the presence of O_2_, pyrolytic products are subsequently oxidized to CO_2_ in the lower oven chamber maintained at 800°C. After passing through the CO_2_ analyzer, the CO_2_ that evolved from three middle reach samples (YB-5, 36MC, and 2605) was further collected downstream in switchable cryogenic traps as temperature-based fraction splits (*30*, *50*). Fractions of CO_2_, once collected by the switchable cryogenic traps, were transferred downstream to the vacuum line, where the CO_2_ was purified and quantified for isotopic measurements.

Throughout the analyses, instantaneous carrier gas flows, pyrolysis oven temperatures, evolved CO_2_ gas concentrations, and the cumulative amount of CO_2_ gas were recorded. Thermograms of normalized CO_2_ concentrations versus temperature were converted and presented in the activation energy mode (*24*). After being purified on a vacuum line, the stable carbon (reported as δ^13^C) and radiocarbon (^14^C activity, reported as Fm) isotopic compositions of the cryogenic-trapped CO_2_ splits were measured at the National Ocean Sciences Accelerator Mass Spectrometry facility. The radiocarbon data, reported as Fm values, were corrected for procedural blanks (see the Supplementary Materials). The precision of the oven ramping rate is <5%, whereas the precision of the CO_2_ concentration measurements is ≍5 ppm (*60*). The precision of the isotopic measurements is better than 0.003 for Fm values and better than 0.1‰ for δ^13^C values.

### Ramped combustion–evolved CO_2_ gas analysis

As described previously (*25*, *31*), the RC-EGA instrument consists of a Netzsch simultaneous thermal analyzer (STA 409PC Luxx) coupled to a LI-840 CO_2_/H_2_O infrared gas analyzer (LI-COR Biosciences, Lincoln, NE). An aliquot of decarbonated, freeze-dried sediment containing 1 mg of OC or <50 mg of sediment, whichever was less, was transferred into a crucible with a pierced lid and loaded onto the autosampler of the STA. After being conditioned at 105°C to remove moisture, samples were heated to 975° to 1000°C at a ramping rate of 5° or 10°C min^−1^. A total carrier gas flow of 40 ml min^−1^ was maintained, with protective nitrogen gas of 10 ml min^−1^ and Ultra-Zero Grade air of 30 ml min^−1^ throughout the whole system. CO_2_ concentrations in the evolved gas were analyzed by the infrared gas analyzer coupled to the outlet of the STA (*31*). Results of RC-EGA have been shown to be highly reproducible, based on repeated analyses of a variety of samples (*25*); thus, analyses on individual samples were not replicated.

### Compilation of published data on sediments

The collection of global sediment thermal decomposition results is based on the recently compiled dataset in (*23*) and further incorporates more recent results (Supplementary Materials). Several modifications are noted. First, data published from the Pan-Arctic Ocean were previously excluded because of protracted inland storage and significant reservoir ages but were included in our discussion of activation energies. Second, lake sediments that were formerly attributed to the particulate OC pool were also included. This reattribution is justified as sediments differ from particulates in that the former are accumulated in sedimentary basins through various depositional processes. The compilation of carbon loading (OC/MSA) data from passive margins was recently updated (*13*); see the Supplementary Materials for details. Likewise, the fjord sediment OC content dataset was updated with previously published data (*12*, *15*).

### Data analysis

Discrepancies were observed between thermograms generated using RPO and RC-EGA (Supplementary Materials). More specifically, *T*_max_, the temperature of maximum CO_2_ evolution for a given sample obtained by the two methods, was offset by a few tens of degrees. The discrepancy likely arises due to temperature monitoring or calibration errors on the RPO system because the thermocouple sensor is mounted outside the quartz reactor. Hence, RPO thermograms were normalized to the RC-EGA data by matching *T*_max_ values of duplicate sample sets. Results of the RPO or RC-EGA analyses were then converted to activation energy distributions in Python (data file S1), as described in (*24*). Thermal decomposition temperatures were also corrected for differences in ramp rates (e.g., 5° versus 10°C). The mean value of *E* (μ*_E_*) and the SD of *E* (σ*_E_*) were calculated following the functions given in (*23*). The fraction of OC within a specific *E* range was calculated as

$$f_{a\;<\;E\;<\;b\;}=\int_{a}^{b}\;p(E)dE$$ (1)

SD values of μ*_E_*, σ*_E_*, and *f*_*a* < *E* < *b*_ were calculated on the basis of duplicate measurements by RC-EGA.

The calculation of fjord mean OC is based on a compiled fjord %OC dataset, which now contains 389 %OC measurements from this and previously published studies (Supplementary Materials). A bootstrap resampling method (*61*) was carried out to better estimate the statistical properties of this dataset. Each resampled data group contains 40 samples, [OC_1_, OC_2_, …, OC_40_], randomly chosen from the 389 measurements on a per-fjord basis, which, after averaging, yields one estimate of the arithmetic mean value of OC

$$\bar{OC}=\frac{1}{40}\sum_{i=1}^{40}{OC}_{i}$$ (2)

After repeating the bootstrap resampling process 1 × 10^4^ times, it generated a dataset of %OC containing 1 × 10^4^ values, [$\${\bar{OC}}_{1}\$$, $\${\bar{OC}}_{2}\$$, …, $\${\bar{OC}}_{1\times 10^{4}}\$$], which, to a large extent, follows a lognormal distribution (*61*). The dataset was further natural log-transformed and displays a normal distribution of (μ_oc,_ σ_oc_^2^), where μ_oc_ and σ_oc_ are the mean and the SD of the transformed ln(%OC) dataset. The mean (OC_mean_) and SD (OC_SD_; symmetrical based on definition) (*61*) values of the global fjord %OC were calculated, respectively, as

$$e^{\mu_{oc}+\frac{\sigma_{\mathrm{oc}}^{2}}{2}}$$ (3)

and

$$\sqrt{(e^{\sigma_{\mathrm{oc}}^{2}}-1)\cdot e^{2\mu_{oc}+\sigma_{\mathrm{oc}}^{2}}}$$ (4)

The 95% CI of the mean fjord %OC value was calculated on the basis of the (%OC) lognormal cumulative distribution function (CDF)

$$F(x)=\frac{1}{2}[1+\mathrm{erf}\left(\frac{\ln x-\mu_{oc}}{\sqrt{2}\sigma_{\mathrm{oc}}}\right)]$$ (5)

The lower and upper bounds (*x* values) of the CI are calculated as values where the *F*(*x*) curve crosses 2.5 and 97.5%. Because the error function erf takes an integrated form, this step does not have an analytical solution and thus is solved numerically (*61*). In this work, the solved mean value and CI bounds are comparable to values calculated using the simple expressions $\$e^{\mu_{oc}}\$$ and $\$e^{\mu_{oc}+2\sigma_{oc}}/e^{\mu_{oc}-2\sigma_{oc}}\$$, respectively.

The “residual” OC (ROC; %OC after glacial decomposition) content of fjord sediments was calculated on the basis of the compiled carbon loading (OC/MSA) data for global passive margin systems (containing 534 measurements) and MSA data of fjord sediments (containing 57 measurements), after acquisition of modeled density distributions. Consistent with the processing of global fjord %OC above, a bootstrap resampling method (*61*) (40 samples each data group, 1 × 10^4^ times resampling) was carried out to acquire the density distribution of the arithmetic mean values of OC/MSA [$\${\bar{OC/MSA}}_{1}\$$, $\${\bar{OC/MSA}}_{2}\$$, …, $\${\bar{OC/MSA}}_{1\times 10^{4}}\$$] and MSA [$\${\bar{MSA}}_{1}\$$, $\${\bar{MSA}}_{2}\$$, …, $\${\bar{MSA}}_{1\times 10^{4}}\$$]. Similar to global fjord %OC, both OC/MSA and MSA display logarithm normal distributions of (μ_OC/MSA,_ σ_OC/MSA_^2^) and (μ_MSA,_ σ_MSA_^2^), in which μ and σ are the mean and SD of the transformed normal distribution, respectively.

ROC is then estimated according to the formula

$$ROC\;[\%]=\frac{100\%}{1000}\frac{OC/MSA\;[\text{mg OC}\;m^{-2}]}{MSA\;[m^{2}\;g^{-1}]}$$ (6)

Forty values of OC/MSA and MSA were randomly generated assuming both follow logarithm normal distributions of (μ_OC/MSA,_ σ_OC/MSA_^2^) and (μ_MSA,_ σ_MSA_^2^). Substituting each value of OC/MSA [OC/MSA_1_, OC/MSA_2_, …, OC/MSA_40_] and MSA [MSA_1_, MSA_2_, …, MSA_40_] into the above equation yields one data group of size 40, [ROC_1_, ROC_2_, …, ROC_40_]. After repeating this process 1 × 10^4^ times, the generated dataset, [$\${\bar{ROC}}_{1}\$$, $\${\bar{ROC}}_{2}\$$, …, $\${\bar{ROC}}_{1\times 10^{4}}\$$], follows a lognormal distribution. The lognormal [ROC] dataset was subsequently transformed to the normal distribution of *N* (μ_ROC_, $\$\sigma_{ROC}^{2}\$$), where μ_ROC_ refers to the mean, and σ_ROC_ refers to the SD of the transformed dataset [ln(ROC)]. Accordingly, the mean (ROC_mean_) and SD (ROC_SD_) of the ROC content were calculated (*61*), respectively, as

$$e^{\mu_{ROC}+\frac{\sigma_{ROC}^{2}}{2}}$$ (7)

and

$$\sqrt{(e^{\sigma_{ROC}^{2}}-1)\cdot e^{2\mu_{ROC}+\sigma_{ROC}^{2}}}$$ (8)

The 95% CI of the returned ROC was calculated on the basis of the CDF function, as described above. The mean and SD of the proportion of degradable OC in fjord sediments are calculated, respectively, as (*62*–*69*)

$$1-\frac{{ROC}_{mean}}{{OC}_{mean}}$$ (9)

and

$$\sqrt{{\left(\frac{ROC_{SD}}{ROC_{mean}}\right)}^{2}+{\left(\frac{OC_{SD}}{OC_{mean}}\right)}^{2}}$$ (10)
