# Supplementary material for: Global fjords as transitory reservoirs of labile organic carbon modulated by organo-mineral interactions
Source: Sci Adv. 2022 Nov 18;8(46):eadd0610. doi: 10.1126/sciadv.add0610 (PMC9674297; doi:10.1126/sciadv.add0610)
Supplement: Supplementary file 1 — Supplementary Text Figs. S1 to S7 Tables S1 to S4 [file sciadv.add0610_sm.pdf]

Science Advances
AAAS

Supplementary Materials for

Global fjords as transitory reservoirs of labile organic carbon modulated by organo-mineral interactions

Xingqian Cui *et al.*

Corresponding author: Xingqian Cui, cuixingqian@sjtu.edu.cn

Sci. Adv. **8**, eadd0610 (2022)
DOI: 10.1126/sciadv.add0610

The PDF file includes:

Supplementary Text
Figs. S1 to S7
Tables S1 to S4

Other Supplementary Material for this manuscript includes the following:

Data file S1

Supplementary Text

Data sources for compiled datasets.

This study updates the thermal decomposition dataset compiled earlier by ref. (23), and includes additional data reported recently by ref. (38, 39, 40, 62, 65, 67). Data from the Arctic Colville River Delta, Simpson Lagoon, and Lake Whittington are also included in the dataset of global thermal decomposition (41, 63, 66). Starting with the compiled dataset on fjord OC contents (12, 15), this study further incorporated recently published data by ref. (33, 64, 68). Carbon loading data on passive margins were reported previously in a few studies (13, 43), to which recently published data (46) were added. It should be noted that our complied datasets may not fully cover all publicly available data; nevertheless, they reflect general trends and patterns within uncertainties.

A comparison of ramped combustion-evolved CO_2 gas analysis (RC-EGA) and ramped pyrolysis oxidation (RPO) thermograms.

Ten samples were analyzed by both RC-EGA and RPO. Some discrepancies were observed between thermograms generated after RPO temperature calibration. In general, thermograms generated from RC-EGA have a better resolution than RPO. Notably, multiple peaks are observed in each RC-EGA thermogram, whereas RPO thermograms commonly only show one humpy peak (Fig. S2). This likely arises for three reasons. First, the dead volume, between CO_2 generation and measurements, is larger in the RPO system, which is not an issue for the well-developed RC-EGA technique. Second, samples were measured under pyrolysis mode on the RPO system, whereas they were run on the oxidation mode on the RC-EGA system. This means that sedimentary organic carbon is in direct contact with free oxygen in the upper chamber of the pyrolysis oven under the oxidation mode, whereas volatized organic carbon is pyrolyzed and oxidized in the lower chamber of the pyrolysis oven under pyrolysis mode. Third, differences in carrier gases composition in the RPO (helium) and RC-EGA (Ultra-Zero air) systems may cause differences in O_2 fugacity. Irrespective, the lower resolution of RPO relative to RC-EGA does not affect our data interpretations. For example, strong correlations are observed between RC-EGA and RPO generated data for both μ_E and σ_E (Fig. S6).

Blank correction of ramped pyrolysis oxidation (RPO) radiocarbon data.

Any manipulation of a sample prior to isotope or chemical analysis can induce contamination, the RPO-^{14}C preparation is not an exception. To determine the mass of procedural blank contamination, which is too small to measure for radiocarbon content by itself, we measured standards of known isotope composition at different masses, assuming that the blank contamination mass is independent of the sample mass. If that assumption is true, smaller samples will fall farther from the true value. This method of blank contamination assessment has been shown to work well for RPO (69), where any type of carbon-bearing material with a known isotope composition can be introduced to the system to measure the effect of blank

contamination. A compilation of all blank mass determinations at USF (Figure S7A) shows that convergence of lines of equal blank contamination mass at high sample mass (to the right in Fig. S7A) have the potential to artificially amplify blank mass uncertainty simply due to the magnitude of analytical uncertainty. Furthermore, two different lots of graphite, used to determine the modern blank, were shown to be different over time. For this project, we used only smaller masses of Ox-I (used to determine dead blank contamination mass) and one lot of graphite (used to determine modern blank contamination mass). Additionally, we used only a three-year window around the time that the samples were measured to reduce the number of blank mass determinations to 10 (Fig. S7B). The net result of the blank contamination correction (Fig. S7C) is miniscule (< 5 μg) for all but the oldest samples. Effectively, the blank mass correction increased the age spectrum of the oldest sample, but had little effect on the other samples.

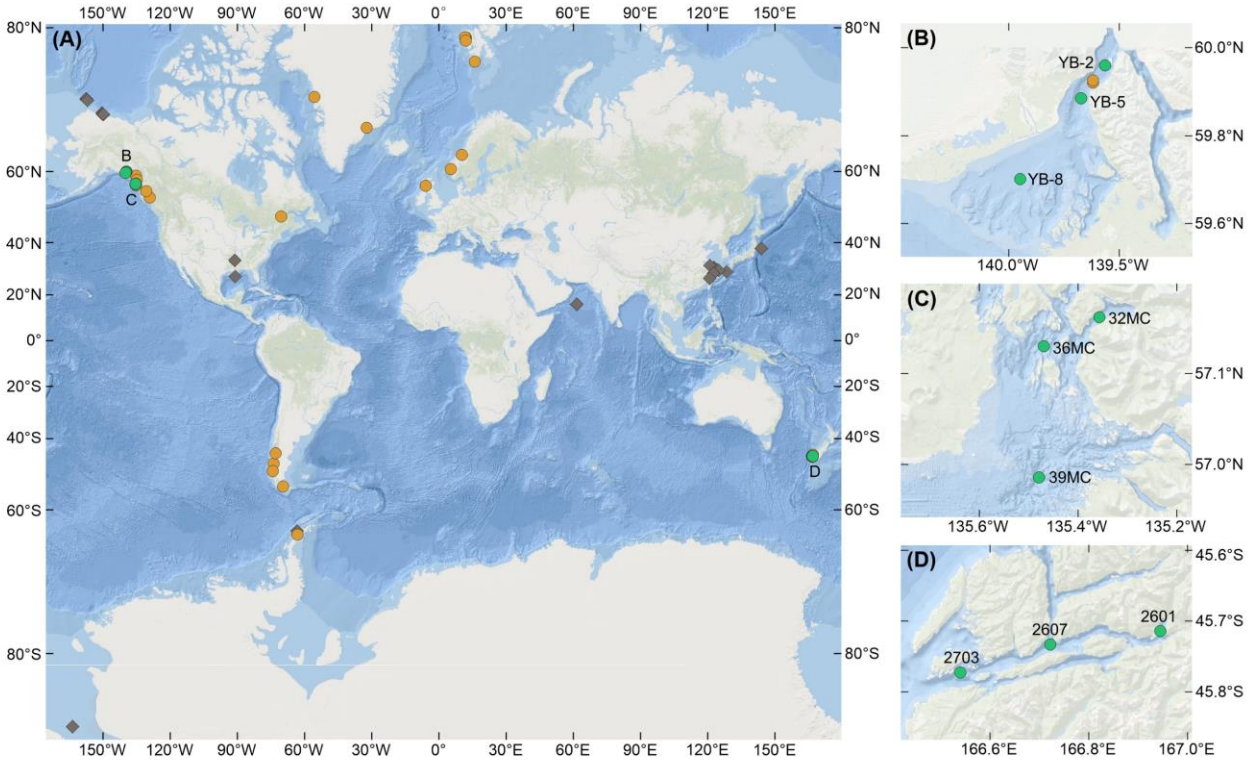

Fig. S1. Global and regional maps showing the location of sediment samples investigated in this study, including fjord sediment samples (dots) and the global sediment sample set (gray diamonds). (A) The global map showing the location of samples analyzed in this study and the compiled global sediment dataset. **(B)**, **(C)**, and **(D)** are enlarged regional plots for three proximal-to-distal transects, including **(B)** the Yakutat Bay, northern SE Alaska, **(C)** the Sitka Sound, southern SE Alaska, and **(D)** the Dusky Sound, Fiordland, New Zealand. Green and orange dots represent all fjord samples, whereas green dots indicate samples further analyzed by ramped pyrolysis oxidation (RPO). The regions where the Yakutat Bay **(B)**, Sitka Sound **(C)**, and Dusky Sound **(D)** are located in the global map and annotated alphabetically in **(A)**.

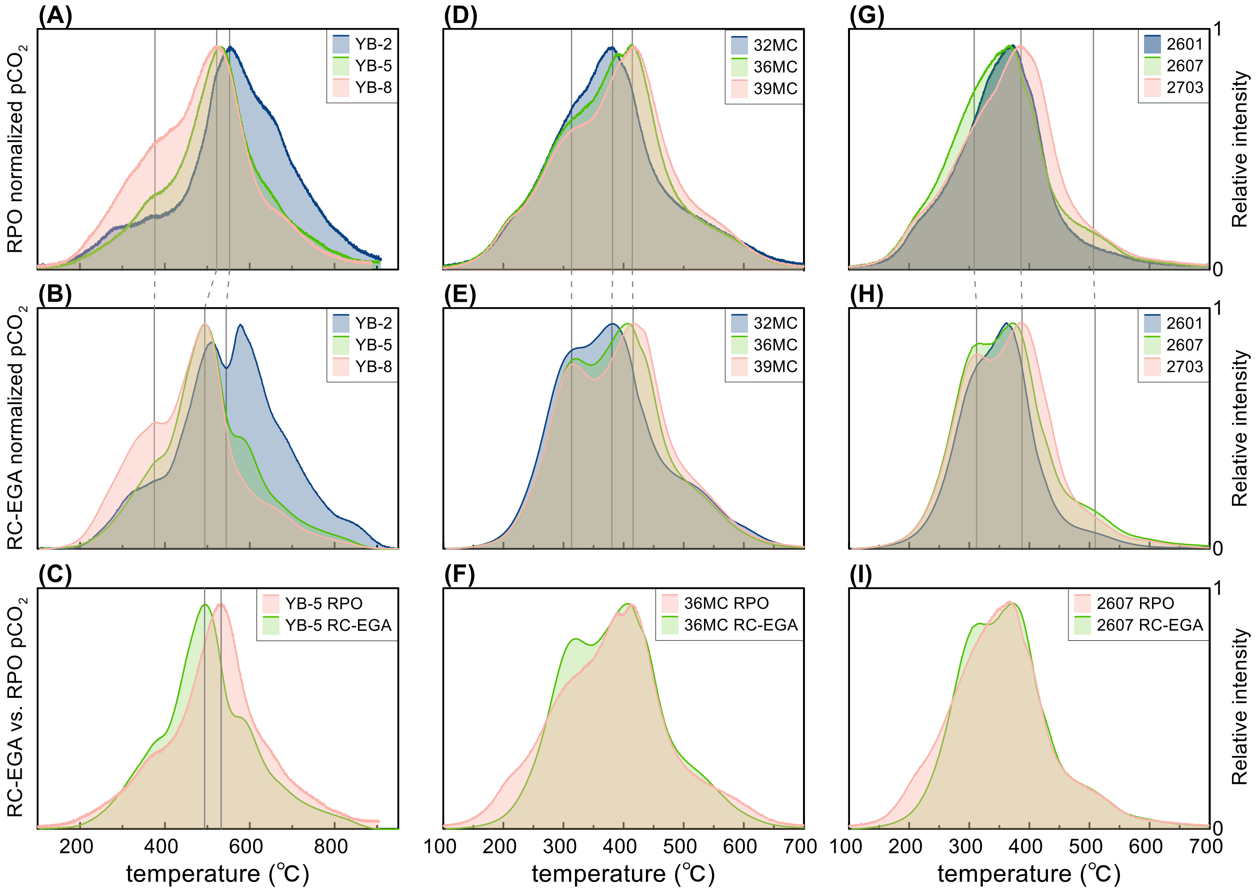

Fig. S2. A comparison of thermograms generated from ramped pyrolysis oxidation (RPO) and ramped combustion-evolved CO_2 gas (RC-EGA) analyses. Samples cover fjord proximal, middle, and distal locations in Yakutat Bay, SE Alaska (proximal: YB-2; middle: YB-5; distal: YB-8), Sitka Sound, SE Alaska (proximal: 32MC; middle: 36MC; distal: 39MC) and Dusky Sound, New Zealand (proximal: 2601; middle: 2607; distal: 2703). These three fjords were chosen to represent the dominance of petrogenic-, marine primary production- and terrestrial-sourced organic carbon. Gray lines represent temperature ties between RPO and RC-EGA thermograms.

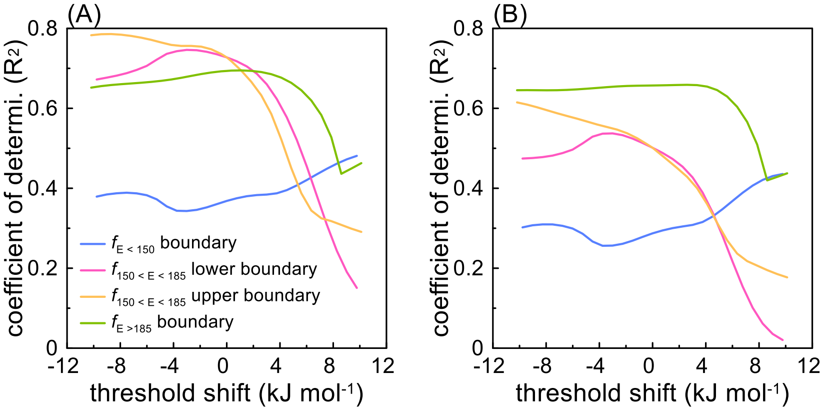

Fig. S3. A sensitivity test to validate the selection of thresholds to separate bulk OC into low-E ($f_{E<150}$), mid-E ($f_{150<E<185}$) and high-E ($f_{E>185}$) fractions. Variations of coefficients of determination (R^2) between three fractions and **(A)** OC content (%OC), or **(B)** OC loading (OC/MSA), when shifting current thresholds by ± 10 kJ mol^{-1}. Results clearly show the highest R^2 values for mid-E and high-E fractions, and the lowest R^2 values for the low-E fraction when threshold shifts are close to 0. This is consistent with the hypothesis that OC with low-E is mostly freely-existing debris, whereas OC with mid-E and high-E is tightly associated with minerals. It is worth noting that the coefficients of determination drop sharply when the lower and upper thresholds (i.e., 150 and 185 kJ mol^{-1}) of the mid-E fraction shift positively, which is explained, respectively, by the narrow E range of the mid-E fraction and the presence of the petrogenic OC (OC_{petro}).

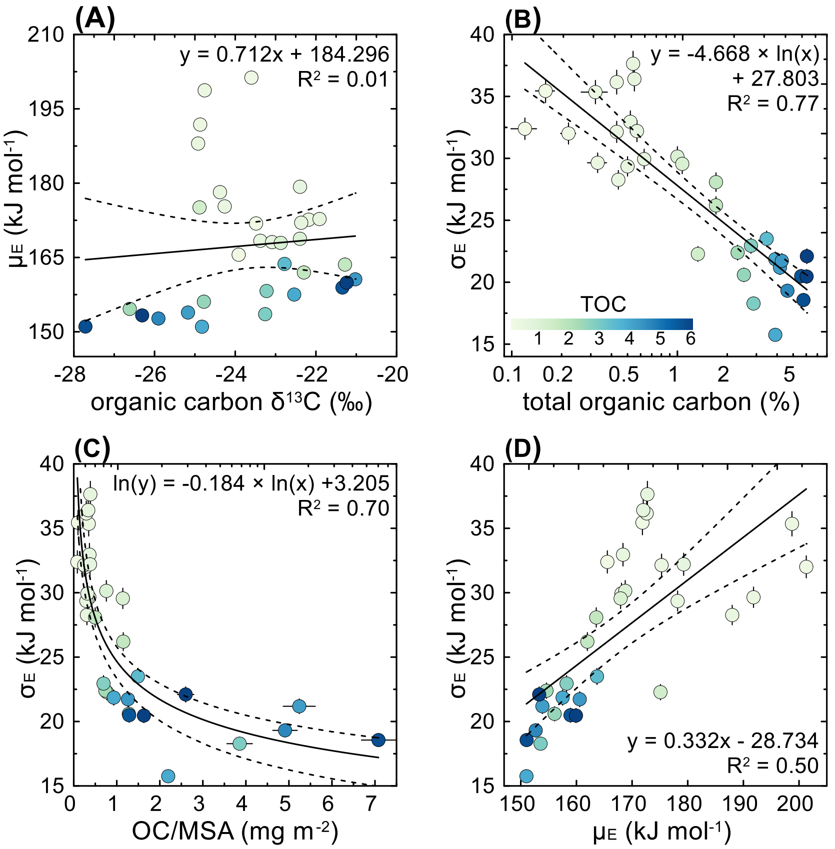

Fig. S4. Correlations between total organic carbon contents, organic carbon $\delta^{13}C$ values, organic carbon loadings (OC/MSA), mean activation energies (μ_E), and standard deviations of the activation energies (σ_E). The colored bar and dots show the range of TOC values. Solid and dashed lines are best fit curves and 95% confidence intervals, respectively.

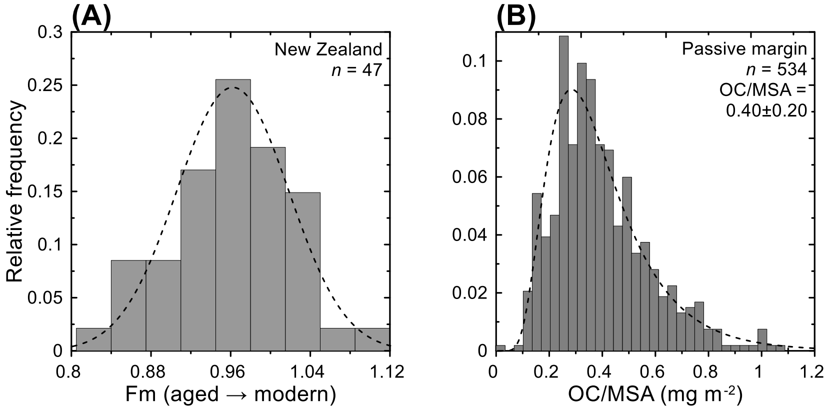

Fig. S5. Supplementary data on radiocarbon fraction modern (Fm) and organic carbon loading (OC/MSA) values. (A) The compiled OC radiocarbon Fm values in the New Zealand fjord sediments (12, 36). The average Fm value of 0.96 under the normal distribution indicates young radiocarbon ages and, accordingly, short transit times of OC from production to deposition in sediments. **(B)** The compiled OC/MSA data of passive margin sediments (13, 43, 46). The data display a logarithmic normal distribution. Data are presented as relative frequencies.

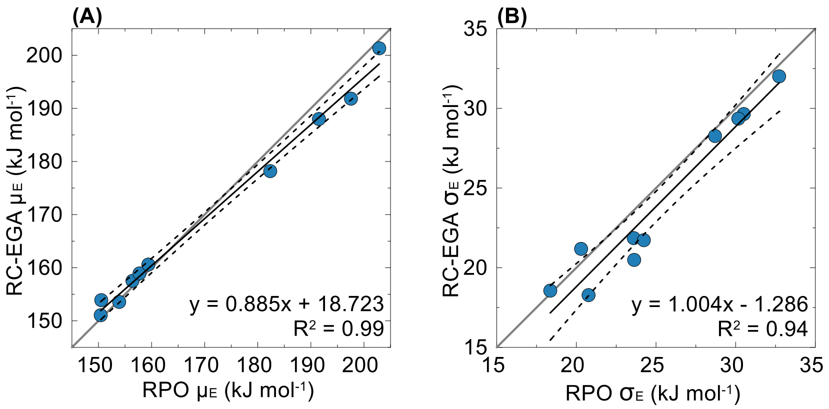

Fig. S6. A comparison of the ramped pyrolysis oxidation (RPO) and ramped combustion-evolved gas analysis (RC-EGA) results in terms of (A) mean (μ_E) and (B) standard deviation (σ_E) of the activation energy. The gray lines represent the 1:1 relationship, whereas solid and dashed black lines represent, respectively, linear regression lines and 95% confidence intervals.

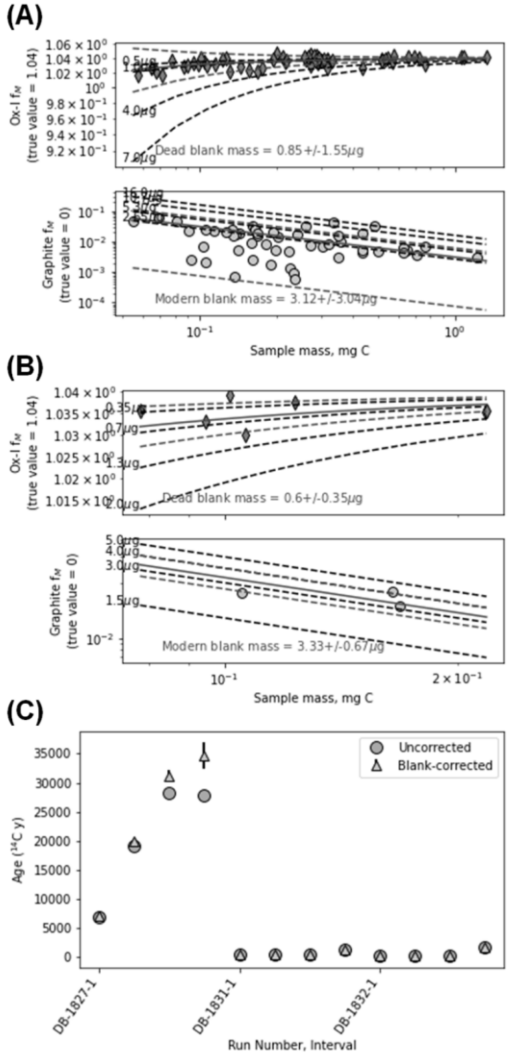

Fig. S7. The blank correction of ramped pyrolysis oxidation (RPO) radiocarbon data. (A) 109 determinations of blank contamination were carried out at USF using the RPO system. We measured the blank contamination as in ref. (69), except that copper and stainless tubing was substituted from the Teflon tubing of the Tulane University system. The result is a lower level of blank contamination. **(B)** To calculate the blank contamination for this project, we only used the Ox-I measurements below 240 micrograms and the measurements from a single lot of graphite. Additionally, we limited the compilation of results to analyses done between 2017 and 2020, when these samples were analyzed. This reduces the number of measurements to 10. **(C)** The effect of blank contamination corrections on these samples is minimal for younger samples, but appreciable for older ones. The net effect is to increase the age spectrum of the oldest samples (≈ 7000 years), whereas the younger samples become modestly older (< 100 years). Run numbers DB-1827, DB-1831 and DB-1832 are lab run numbers and correspond to samples YB-5, 36MC and 2607, respectively.

Table S1. Acronyms, abbreviations and indices used in this study.

Abbreviations Acronyms, indices	Definition and explanation
OC_{bio}	Biospheric organic carbon, including terrigenous (OC_{terr}) and marine (OC_{mari}) organic carbon; Any type of organic carbon produced through biological activities.
OC_{terr}	Terrigenous organic carbon; Organic carbon that is produced through contemporary biological activities, such as vascular plants, organic debris and soil OC.
OC_{mari}	Marine organic carbon; Organic carbon produced mostly by marine primary producers.
OC_{petro}	Petrogenic (or fossil) organic carbon; chiefly organic carbon from sedimentary bedrock erosion.
RC-EGA	Ramped combustion-evolved CO_2 gas analysis; A commercially available instrument for organic carbon thermal decay analysis.
RPO-^{14}C	Ramped pyrolysis oxidation-^{14}C analysis; In-lab assembled facility for OC thermal decay and fractional isotopic analysis.
E	Activation energy (of OC; kJ mol^{-1} OC); Inversely modeled from thermal decay of OC.
p(E)	The distribution of E across an E range (typically 0 - 400 kJ mol^{-1}), calculated based on the Python package "rampedpyrox", reported in the form of relative frequency, with the unit of mol kJ^{-1}.
μ_E	The mean activation energy of OC in a particulate sample, calculated based on the distribution of the activation energy of OC.
fractional μ_E	The mean activation energy of OC collected in a specific temperature range (e.g., 300 - 500 ˚C), modeled based on the Python package "rampedpyrox".
σ_E	The standard deviation of the distribution of activation energies [p(E)].
$f_{E < 150}$	The fraction of OC in a given sample that is decomposed thermochemically with activation energies of < 150 kJ mol^{-1}.
$f_{150< E < 185}$	The fraction of OC in a given sample that is decomposed thermochemically with activation energies in the range of 150 - 185 kJ mol^{-1}.
$f_{E > 185}$	The fraction of OC in a given sample that is decomposed thermochemically with activation energies of > 185 kJ mol^{-1}.
MSA	Mineral surface area of solid particles in a sample (e.g., soils, sediments, suspended particulate matter)
OC/MSA	Organic carbon loading, defined as the density of OC per unit surface area

Table S2. Sampling location, sample layer, water depth and coordinates of samples in this study.

Sample ID	Region	Fjord	Sample Layer	Water Depth(m)	Latitude	Longitude
Svl-11	Svalbard	Kongsfjorden	0-1 cm	310	78.94945	11.98485
Svl-15	Svalbard	Hornsund	0-1 cm	107	77.0116	16.01548
Svl-37	Svalbard	Krossfjord	0-1 cm	165	79.20771	12.05579
Svl-39	Svalbard	Krossfjord	0-1 cm	250	79.23625	11.695
GLW-1	Greenland	Upernavik Fjord	Surface	1000	72.94517	-55.6205
GLE-2	Greenland	Kangerlugssuaq Fjord	Surface	730	68.42	-32.276
NW-316	Norway	Trondheimsfjord	0-1 cm	483	63.5072	10.26806
NW-10	Norway	Osterfjorden	Surface	542	60.53448	5.298083
Scot-3	Scotland	Loch Sunart	0-1 cm	58	56.66898	-5.86328
YB-2	SE Alaska	Yakutat Bay	Surface	176	59.95983	-139.564
YB-3	SE Alaska	Yakutat Bay	0-1 cm	242	59.92596	-139.619
YB-4	SE Alaska	Yakutat Bay	Surface	246	59.92067	-139.619
YB-5	SE Alaska	Yakutat Bay	Surface	254	59.88517	-139.673
YB-8	SE Alaska	Yakutat Bay	Surface	122	59.70133	-139.948
LC-3	SE Alaska	Lynn Canal	grab	193	59.06222	-135.336
56MC	SE Alaska	Cross Sound	3-5 cm	607	58.11168	-134.923
32MC	SE Alaska	Sitka Sound	0-1 cm	141	57.16182	-135.357
36MC	SE Alaska	Sitka Sound	0-1 cm	133	57.13002	-135.469
39MC	SE Alaska	Sitka Sound	0-1 cm	216	56.9856	-135.479
19MC	SE Alaska	Crawfish Inlet	0-1 cm	109	56.75185	-135.182
WS-2	SE Alaska	Wilson Arm	grab	152	55.33778	-130.683
DC-6	B. Columbia	Douglas Channel	Surface	400	53.67427	-129.122
SF-8	E. Canada	Saguenay Fjord	0-5cm	261	48.357	-70.451
2401	New Zealand	Doubtful Sound	1-2 cm	435	-45.3194	166.9802
2601	New Zealand	Dusky Sound	1-2 cm	83	-45.7143	166.9451
2607	New Zealand	Dusky Sound	1-2 cm	309	-45.7334	166.7223
2703	New Zealand	Dusky Sound	1-2 cm	195	-45.7728	166.5399
2903	New Zealand	Long Sound	1-2 cm	336	-45.9833	166.8191
PF-3	Patagonia	Puyuhuapi Fjord	5-6 cm	260	-44.8247	-72.9345
BC-4	Patagonia	Baker Channel	0-2 cm	1058	-47.989	-73.827
EF-4	Patagonia	Europa Fjord	0-2 cm	435	-50.1853	-74.2105
AF-3	Patagonia	Anisworth Fjord	0-2 cm	581	-54.3837	-69.5871
AP-4	Antarctica	Flandres Bay	0-5 cm	650	-65.097	-63.17

Table S3. Bulk measurements, surface area, and activation energies of sedimentary organic carbon investigated in this study. μ_E and σ_E indicate mean and standard deviation of the activation energy. $f_{E<150}$, $f_{150<E<185}$, and $f_{E>185}$ refer to fractions of OC within E ranges of <150, 150-185, and >185 kJ mol^{-1}.

Sample ID	TOC	$\delta^{13}C$	MSA $m^2\ g^{-1}$	OC/MSA mg m^{-2}	μ_E kJ mol^{-1}	σ_E kJ mol^{-1}	$f_{E<150}$	$f_{150<E<185}$	$f_{E>185}$
Svl-11	1.07	-22.87	9.38	1.14	167.9	29.6	0.30	0.41	0.29
Svl-15	1.33	-24.89	17.30	0.77	175.1	22.3	0.15	0.40	0.45
Svl-37	0.54	-21.91	14.01	0.39	172.8	37.6	0.33	0.37	0.30
Svl-39	0.55	-22.37	16.32	0.34	172.0	36.4	0.31	0.38	0.31
GLW-1	0.16	-23.48	16.29	0.10	171.9	35.4	0.32	0.39	0.30
GLE-2	0.12	-23.92	14.30	0.08	165.6	32.4	0.38	0.36	0.26
NW-316	1	-22.40	13.20	0.76	168.8	30.1	0.29	0.42	0.28
NW-10	3.46	-22.78	23.21	1.49	163.7	23.5	0.29	0.50	0.20
Scot-3	1.71	-22.30	14.86	1.15	162.0	26.2	0.35	0.42	0.23
YB-2	0.22	-23.60	7.81	0.28	201.3	32.0	0.07	0.21	0.72
YB-3	0.33	-24.87	9.09	0.36	191.8	29.6	0.09	0.22	0.70
YB-4	0.32	-24.76	9.21	0.35	198.7	35.3	0.08	0.25	0.66
YB-5	0.44	-24.92	14.35	0.31	188.0	28.3	0.08	0.36	0.56
YB-8	0.5	-24.38	17.05	0.29	178.2	29.4	0.17	0.40	0.42
LC-3	0.43	-22.18	14.78	0.29	172.6	36.2	0.35	0.29	0.36
56MC	1.71	-21.28	34.33	0.50	163.6	28.1	0.35	0.41	0.24
32MC	3.9	-22.54	41.93	0.93	157.5	21.9	0.38	0.48	0.13
36MC	5.59	-21.34	43.41	1.29	158.9	20.5	0.34	0.54	0.12
39MC	4.27	-21.02	33.90	1.26	160.6	21.7	0.33	0.53	0.14
19MC	6.03	-21.24	37.01	1.63	159.9	20.5	0.33	0.56	0.11
WS-2	3.9	-24.82	17.77	2.19	151.0	15.8	0.46	0.49	0.05
DC-6	2.78	-23.22	40.19	0.69	158.2	22.9	0.38	0.47	0.15
SF-8	2.31	-26.62	31.42	0.74	154.6	22.4	0.47	0.41	0.12
2401	4.62	-25.90	9.40	4.91	152.7	19.3	0.44	0.49	0.07
2601	5.77	-27.71	8.15	7.08	151.1	18.6	0.46	0.49	0.05
2607	4.16	-25.17	7.94	5.24	153.9	21.2	0.44	0.47	0.08
2703	2.88	-23.26	7.48	3.85	153.6	18.3	0.41	0.53	0.06
2903	6.05	-26.31	23.26	2.60	153.3	22.1	0.44	0.49	0.07
PF-3	2.52	-24.78	19.69	1.28	156.1	20.6	0.40	0.48	0.12
BC-4	0.43	-24.26	17.73	0.24	175.3	32.1	0.29	0.30	0.41
EF-4	0.57	-22.39	15.11	0.38	179.3	32.2	0.24	0.27	0.49
AF-3	0.52	-23.38	14.25	0.36	168.4	33.0	0.35	0.34	0.31
AP-4	0.63	-23.09	19.88	0.32	168.1	29.9	0.31	0.39	0.30

Table S4. Ramped pyrolysis oxidation fraction-specific temperature breaks, masses, mean activation energies (μ_E), and isotopic compositions ($\delta^{13}C$, Fm) of the evolved CO_2 after blank correction.

Sample fractions	Temp. break	Mass (μg C)	μ_E±std (kJ mol^{-1})	$\delta^{13}C$±std (VPDB)	Fm±std
YB5-1	248	99.7	146.5±15.5	-25.08±0.17	0.41±0.004
YB5-2	468	207.7	184.5±11.4	-26.05±0.15	0.08±0.002
YB5-3	581	159.9	204.3±10.0	-25.59±0.15	0.02±0.002
YB5-4	761	94.2	234.5±14.0	-21.13±0.16	0.01±0.004
36MC-1	172	60.6	115.7±9.3	-21.92±0.17	0.94±0.004
36MC-2	301	199.4	139.1±9.4	-21.32±0.15	0.94±0.002
36MC-3	421	377.3	165.0±10.3	-21.03±0.15	0.95±0.002
36MC-4	585	82.2	199.6±11.6	-22.39±0.16	0.85±0.003
2607-1	172	79.4	116.8±9.1	-25.13±0.16	0.99±0.004
2607-2	301	305.7	139.6±9.1	-25.45±0.15	0.97±0.002
2607-3	412	349.5	159.9±9.5	-24.98±0.15	0.97±0.002
2607-4	594	77.2	193.1±12.1	-24.94±0.17	0.82±0.003
